# The predictive value of intestinal ultrasound for treatment response in inflammatory bowel disease: a systematic review and pooled data analysis

**DOI:** 10.1093/ecco-jcc/jjag017

**Published:** 2026-04-15

**Authors:** Johanna M B W Vos, Christoph Teichert, Floris A E De Voogd, Maarten J Pruijt, Faridi S Jamaludin, Geert R A M D’Haens, Marc A Benninga, Bart G P Koot, Krisztina B Gecse

**Affiliations:** Department of Paediatric Gastroenterology and Nutrition, Emma Children’s Hospital, Amsterdam UMC, University of Amsterdam, Amsterdam, The Netherlands; Amsterdam Gastroenterology Endocrinology Metabolism Research Institute, Amsterdam UMC, Amsterdam, The Netherlands; Amsterdam Reproduction and Development Research Institute, Amsterdam UMC, Amsterdam, The Netherlands; Amsterdam Gastroenterology Endocrinology Metabolism Research Institute, Amsterdam UMC, Amsterdam, The Netherlands; Department of Gastroenterology and Hepatology, Amsterdam UMC, Amsterdam, The Netherlands; Department of Gastroenterology and Hepatology, Amsterdam UMC, Amsterdam, The Netherlands; Amsterdam Gastroenterology Endocrinology Metabolism Research Institute, Amsterdam UMC, Amsterdam, The Netherlands; Department of Gastroenterology and Hepatology, Amsterdam UMC, Amsterdam, The Netherlands; Amsterdam University Medical Centre, University of Amsterdam, Medical Library AMC, Amsterdam, The Netherlands; Department of Gastroenterology and Hepatology, Amsterdam UMC, Amsterdam, The Netherlands; Department of Paediatric Gastroenterology and Nutrition, Emma Children’s Hospital, Amsterdam UMC, University of Amsterdam, Amsterdam, The Netherlands; Department of Paediatric Gastroenterology and Nutrition, Emma Children’s Hospital, Amsterdam UMC, University of Amsterdam, Amsterdam, The Netherlands; Department of Gastroenterology and Hepatology, Amsterdam UMC, Amsterdam, The Netherlands

**Keywords:** predictive value, intestinal ultrasound, inflammatory bowel disease

## Abstract

**Introduction:**

Intestinal ultrasound (IUS) is a promising tool for predicting treatment response in inflammatory bowel disease (IBD). We aimed to systematically review existing literature on the predictive value of IUS for treatment response.

**Methods:**

A literature search was performed until May 2025. We included articles assessing treatment response in IBD using IUS. Data were analyzed separately for Crohn’s Disease (CD), ulcerative colitis (UC) and acute severe ulcerative colitis (ASUC). Pooled data analysis was performed for bowel wall thickness (BWT) in CD patients starting anti-TNF (tumor necrosis factor).

**Results:**

In total, 31 articles were included (18 CD, 9 UC, and 4 ASUC). In 8/10 CD studies, IUS at week 4-8 could distinguish future responders, with BWT change ranging from −43% to −14.6% in responders vs. −14% to + 2% in nonresponders. In the pooled data analysis of anti-TNF-treated CD patients (*n* = 236) a 23% decrease from baseline at week 4-8 (area under the receiver operating characteristic curve [AUROC] 0.82) and 27% decrease at week 12-16 (AUROC 0.78) could predict future response. In 2/2 UC studies, IUS after 4-8 weeks could predict future endoscopic remission vs. non-remission with a decrease in BWT ranging from −55% to −49% vs. −38% to −17% respectively. In ASUC, change in BWT after 1-3 days could predict need for salvage therapy (−34% vs. −10%).

**Conclusions:**

Intestinal ultrasound predicts response at an early stage after treatment initiation, both in CD as well as in UC. In anti-TNF-treated CD patients, 23% and 27% decrease in BWT after 4-8 weeks and 12-16 weeks of treatment initiation predicts outcomes.

## 1. Introduction

Inflammatory bowel disease (IBD)—which includes Crohn’s Disease (CD) and ulcerative colitis (UC)—is a chronic disease of the gastro-intestinal tract with a relapsing and remitting course.^1^^,^^2^ The treatment poses significant challenges as there are no predictive markers of treatment response and therefore choice of treatment is often based on a trial-and-error approach. Choosing the right treatment in an early stage of disease is pivotal to prevent disease progression and long-term complications.^3^

Clinical symptoms often do not accurately reflect active inflammation.^4^ Therefore, adjustments in pharmacologic treatment should be based on objective markers of inflammation. Fecal calprotectin correlates well with disease activity but lacks information about the disease location and extent.^5^^,^^6^ Mucosal healing (MH), assessed by endoscopy, is the gold standard to determine inflammation in IBD. Multiple studies demonstrated MH as a strong predictor for improved long-term outcomes. Nevertheless, endoscopy is invasive and expensive, and inflammation in IBD does not only affect the mucosa. Recently, transmural healing (TH) as evaluated by cross-sectional imaging has been associated with better long-term outcomes.^7–9^ Computed tomography and magnetic resonance imaging are less suitable for monitoring in IBD due to availability, costs, radiation exposure and they are in general poorly tolerated by children.^10^

Intestinal ultrasound (IUS) has become a commonly used non-invasive, point-of-care, radiation-free imaging modality for monitoring IBD activity, including assessing TH. Recent studies have shown that IUS correlates well with endoscopy, and inflammatory markers, such as fecal calprotectin and C-reactive protein.^6^^,^^11^ As this imaging modality is easy and cost-efficient to repeat after treatment initiation, it could be used to predict early treatment response. This is underscored by the recently published ECCO-ESGAR-ESP-IBUS guideline, which recommends IUS as one of the modalities for evaluating treatment response at approximately 12 weeks.^12^ However, the relevance of different IUS parameters, the thresholds, and their predictive values are not specified. Therefore, the aim of this systematic review is to provide an overview of the current literature and to evaluate the predictive value of B-mode IUS parameters for treatment response in IBD.

## 2. Methods

The study protocol was registered with the International Prospective Register of Systematic Reviews (PROSPERO) with CRD 42024497503. We followed the methodology for conducting and reporting a systematic review, as described by the Preferred Reporting Items for Systematic Reviews and Meta-analysis (PRISMA) statement.^13^

### Data sources and searches

Published studies were identified using Pubmed, Medline (Ovid), Embase (Ovid), Cochrane Central Register of Controlled Trials (CENTRAL) and Cinahl (Ebsco) from inception until the May 22, 2025 for each database. The search strategy for PubMed is outlined in Supplementary Material 1.

### Study eligibility criteria

Studies were included if they involved prospective studies of B-mode IUS in IBD patients starting treatment, that described the predictive value of IUS for treatment response, regardless of sample size. Studies were excluded if they did not include IBD patients, were not written in English, no full text was available, were animal studies, no therapy was started, studied only advanced IUS modalities other than B-mode or if IUS was not performed prior to the study endpoint.

Two authors (J.V., C.T.) independently reviewed study titles and abstracts, followed by full text assessment for eligibility. In case of disagreement, the article was included for the full text analysis. In the next phase, the full text of all selected articles was analyzed. Disagreement was resolved by consensus. If full text was not available, corresponding authors were contacted once to retrieve the full text article.

### Data extraction and quality assessment

Extracted data included study characteristics (author, year of publication), clinical characteristics (study population, treatment(s), time point(s) of IUS, definition of response, outcome measures and results [bowel wall thickness and other parameters]). If the data allowed the decrease (%) in bowel wall thickness (BWT) compared to baseline was calculated and added to the table.

To assess the quality of the included studies the Quality in Prognostic Studies (QUIPS) tool was used.^14^  Both reviewers (J.V., C.T.) separately evaluated the studies. Disagreement was resolved by discussion to reach a consensus. The following six domains were assessed: study participation, study attrition, prognostic factor measurement, outcome measurement, study confounding, and statistical analysis and reporting. The risk of bias was categorized for each domain as low, moderate or high risk of bias. Scoring is specified in Supplementary Material 2.

### Study classification

Study populations were stratified for CD, UC, or acute severe ulcerative colitis (ASUC) and subsequently for IUS time-points. Time-points for CD and UC were baseline (week 0); rapid (<4 weeks), short-term (4-8 weeks), intermediate (12-16 weeks) and long-term (>16 weeks). Acute severe ulcerative colitis was not stratified for timepoints.

Study outcome measures to define responders were categorized based on (1) Endoscopy (defined by endoscopic scores); (2) Therapeutic (defined by the need for treatment intensification, hospitalization, and/or surgery); (3) Clinical (defined by clinical scores) and (4) Sonographic (defined by IUS parameters). For the definitions used by each study, see Table S1.

### Pooled data analysis

Pooled data analysis was only conducted for anti-TNF (tumor necrosis factor) treatment in CD. Authors of studies which included at least five anti-TNF CD patients were contacted to obtain raw data on BWT and treatment response status (responder or nonresponder as defined in their publication). In case no response from authors was received, the study was excluded from this sub-analysis.

To determine a clinically relevant cutoff value for BWT, we aimed for a specificity threshold of 90%, prioritizing a high true negative rate in clinical decision-making to prevent unnecessary treatment switch. Additionally, the Youden index was used to identify the cutoff value that maximizes the sum of sensitivity and specificity.

### Statistical analysis

Statistical analysis of the pooled data was performed with SPSS Statistics for Windows, version 28 [IBM Corp., Armonk, NY, USA]. All normally distributed data were reported in mean ± standard deviation [SD] and non-normally distributed in median and interquartile range (IQR). An independent-samples T-test was used to compare continuous variables and a chi square test for dichotomous variables. Area under the receiver operating characteristic curve (AUROC), sensitivity, specificity, optimal cutoff determined by the Youden index, positive predictive value (PPV), and negative predictive value (NPV) were calculated. A *P*-value of ≤0.05 was considered statistically significant.

## 3. Results

### Study inclusion

**Figure 1.**
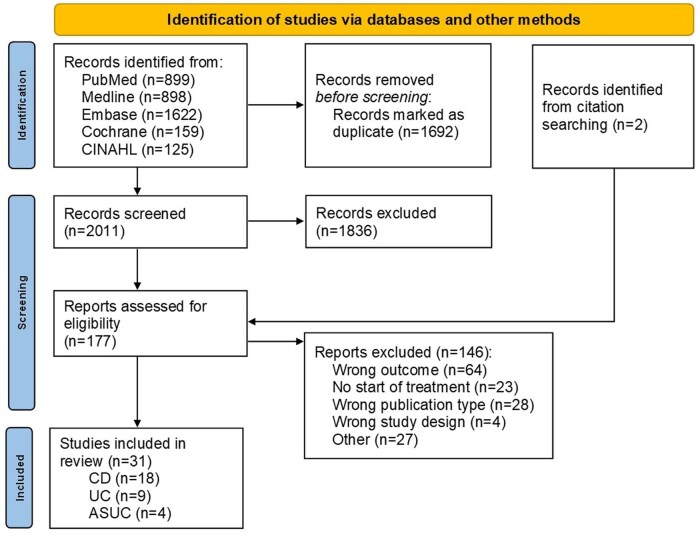
PRISMA flowchart.

A total of 3703 studies were identified and after removing duplicates, 2011 studies remained. Two additional articles were identified from reviewing references from other papers. In total, 1836 articles were excluded following screening of title and abstract, leaving 175 articles. Reasons for exclusion during full text screening are depicted in Figure 1, ultimately resulting in 31 included articles. Of these studies 18 were in CD, 9 in UC and 4 in ASUC. Only 2 studies included children with IBD.

### Prognostic value of IUS in CD

#### Baseline IUS (before treatment start)

Thirteen studies described the predictive value of IUS at baseline (Table 1).^15–27^ Eight studies reported no differences in pretreatment IUS parameters between responders and nonresponders for short- and long-term outcomes.^15^^,^^17^^,^^19^^,^^22^^,^^24–27^ Four studies observed lower baseline BWT in endoscopic responders ranging from week 14-56, with differences ranging from 0.6 mm up to 1.6 mm.^16^^,^^18^^,^^20^^,^^21^ One study only including patients with small bowel strictures described a higher baseline BWT in clinical responders after one year (7.2 mm vs. 6.1 mm).^23^

**Table 1.** Overview of current available studies assessing the predictive value of intestinal ultrasound for treatment response in Crohn’s disease.

IUS at baseline Crohn’s disease							
Author	Treatment	Outcome measurement		Results bowel wall thickness (BWT)		*P*-value	Other IUS results
		Type	Time point	Responders	Nonresponders		
**Laterza^22^**	IFX (*n* = 54)	Both clinical and endoscopic response	Week 12	6.8 ± 0.6 mm (mean ± SD)	6.8 ± 0.6 mm (mean ± SD)	ns	
**Chen^18^**	IFX (*n* = 30)	Both clinical and endoscopic response	Week 14	6.9 ± 1.5 mm (mean ± SD)	8.5 ± 2.3 mm (mean ± SD)	*P* = .027	CDS and BWS: *P* = ns
**Cerna^17^**	IFX sc (*n* = 32)	Therapeutic	Week 30	*Not reported*	*Not reported*		IUS score 6.5 vs. 7.95 *P* = ns
**Huang^21^**	IFX (*n* = 129)	Endoscopic healing	Week 44-56	5.9 mm (mean)	6.6 mm (mean)	*P* <.05	Logistic regression BWS: *P* = 0019 CDS: *P* = .006 i-fat: *P* <.001 IBUS-SAS: 57.6 vs. 71.2 *P* <.01
		**Sonographic transmural healing**		5.9 mm (mean)	6.5 mm (mean)		Logistic regression BWS: *P* = .034 CDS: *P* = ns i-fat: *P* = .001 IBUS-SAS: 55.3 vs. 70.4 *P* <.01
**Orlando^24^**	IFX (*n* = 30)	Therapeutic	Week 52	7.0±1.2 mm (mean ± SD)	5.4±1.5 mm (mean ± SD)	ns	CDS: *P* = ns BWS: *P* = ns
**Lovett^23^**	ADL high dose + thiopurine (*n* = 52) ADL monotherapy (*n* = 25)	Clinical (obstructive symptom score)	Week 52	7.2 [6.3-7.4] mm (median, [IQR])	6.1 [4.5-7.1] mm (median, [IQR])	*P* = .03	
**De Voogd^19^**	IFX (*n* = 28) ADL (*n* = 12)	Endoscopic response	Week 12-34	4,7 [4,2-6,8] mm (median [IQR])	5,4 [4,7-7,0] mm (median [IQR])	ns	
**Quaia^25^**	ADL (*n* = 55) ADL + CS (*n* = 20) IFX (*n* = 15) IFX + CS (*n* = 25)	Endoscopic response	Week 10-18	7 ± 3 mm (mean ± SD)	7 ± 3 mm (mean ± SD)	ns	
**Saevik^26^**	CS (*n* = 9) ADL (*n* = 1) IFX (*n* = 1) ADL + CS (*n* = 2) IFX + CS (*n* = 1)	Clinical remission and therapeutic	Week 52	*Not reported*	*Not reported*	ns	
**Dolinger^20^**	IFX (*n* = 14) ADL (*n* = 9) UST (*n* = 21)	Endoscopic remission	Week 52	4.4 [4.0-5.2] mm (median [IQR])	5.9 mm [5.3-6.3] mm (median [IQR])	*P* = .004	CDS, BWS and LN: *P* = ns i-fat: *P* = .01 IBUS-SAS: 44 vs. 83 *P* = .006
**Smith^27^**	VEDO (*n* = 8) IFX (*n* = 7) UST (*n* = 6) ADL (*n* = 1) Thiopurine (*n* = 1)	Therapeutic	Week 46	5.2 mm (median)	5.1 mm (median)	ns	CDS, BWS, i-fat and LN: *P* = ns
**Ainora^15^**	UST (*n* = 52)	Endoscopic response	Week 48	*Not reported*	*Not reported*	ns	BWS: *P* = ns
		Endoscopic remission					
**Allocca^16^**	ADL (*n* = 40) IFX (*n* = 8) UST (*n* = 40) VEDO (*n* = 5)	Endoscopic remission	Week 52	*Not reported*	*Not reported*		BUS score: 5.33 vs. 6 *P* = .013

Rapid (<4weeks) Crohn’s disease								
Author	Treatment	Outcome measurement		Time point IUS	Results BWT		P value	Other IUS results
		Type	Timepoint		Responders	Nonresponders		
**Chen^18^**	IFX (*n* = 30)	Both clinical and endoscopic response	Week 14	Week 2	5.0 ± 1.1 mm (mean ± standard deviation [SD]) **−Δ28%** (*P* <.01)	7.9 ± 2.5 mm (mean ± SD) **−Δ7%** (*P* = .638)	*Not reported*	CDS: −Δ0.6mLimberg (*P* <.001) vs. −Δ0.3mLimberg *P* = ns
**Laterza^22^**	IFX (*n* = 54)	Both clinical and endoscopic response	Week 12	Week 2	6.6 mm (mean) **−Δ3.5%**	6.8 mm (mean) **−Δ0.5%**	*P* = .009	
		Clinical relapse after initial response	Week 26	Week 2	6.8 mm (mean) **−Δ4.7%**	6.8 mm (mean) **−Δ3.1%**	ns	
**Smith^27^**	VEDO (*n* = 8) IFX (*n* = 7) UST (*n* = 6) ADL (*n* = 1)Thiopurine (*n* = 1)	Therapeutic	Week 46	Week 2	*Not reported*	*Not reported*	ns	

Short-term (4-8 weeks) Crohn’s disease								
Author	Treatment	Outcome measure		Timepoint IUS	Results BWT		*P*-value	Other IUS results
		Type	Time point		Responders	Nonresponders		
**Laterza^22^**	IFX (*n* = 54)	Both clinical and endoscopic response	Week 12	Week 6	5.8 mm (mean) **−Δ14.6% **	6.8 mm (mean) **−Δ0.5%**	*P* <.001	
		Clinical relapse after initial response	Week 26	Week 6	6.0 mm (mean) **−Δ11.8%**	5.8 mm (mean) **−Δ15.3%**	ns	
**Chen^18^**	IFX (*n* = 30)	Both clinical and endoscopic response	Week 14	Week 6	4.6 ± 1.4 mm (mean ± SD) **−Δ33%** (*P* <.001)	7.5 ± 2.6 mm (mean ± SD) **−Δ12%** (*P* = .370)		CDS: −Δ 1.1 mLimberg *P* <.001 vs. −Δ0.5 mLimberg *P* = ns
**De Voogd^19^**	IFX (*n* = 28) ADL (*n* = 12)	Endoscopic response	week 12-34	Week 4-8	3.9 (2.5-4.8) mm (median [IQR]) **−Δ17%**	5.4 (3.4-6.4) mm (median [IQR]) **Δ0%**	*P* = .005	CDS: −1 point was associated with endoscopic response (OR 2.89, *P* = .039) BWS: *P* = ns Haustrations: 14% vs. 66% LN: *P* = ns i-fat: *P* = ns Motility: *P* = ns
**Saevik^26^**	CS (*n* = 9) ADL (*n* = 1) IFX (*n* = 1) ADL + CS (*n* = 2) IFX + CS (*n* = 1)	Clinical remission and therapeutic	Week 52	Week 4	*Not reported*	*Not reported*	ns	Muscularis propria lower in responders vs. nonresponders (no data mentioned, *P* = .008)
**Quaia^25^**	ADL (n = 55) ADL + CS (*n* = 20) IFX (*n* = 15) IFX + CS (*n* = 25)	Endoscopic response	Week 10-18	Week 6	4 ± 2 mm (mean) **−Δ43%**	6 ± 2 mm (mean) **−Δ14%**	*Not reported*	
**Ainora^15^**	UST (*n* = 52)	Endoscopic response	Week 48	Week 8	**Δ0%** IQR [−14.9; 0.0]	**Δ0%** IQR [−14.3; 0.0]	ns	CDS: *P* = ns
		Endoscopic remission			**Δ0%** IQR [−14.2; 0.0]	**−Δ12.5%** IQR [−15.5; 0.0]	ns	
**Kucharzik^11^**	UST (*n* = 77)	Endoscopic response	Week 48	Week 4	*Not reported*	*Not reported*		−Δ≥25% BWT results in: PPV 22.2 and NPV 73.3
				Week 8				−Δ≥25% BWT results in: PPV 50.0 and NPV 80.0
**Dolinger^20^**	IFX (*n* = 14) ADL (*n* = 9) UST (*n* = 21)	Endoscopic remission	Week 52	Week 8	2.9 [2.3-3.2] mm (median [IQR]) **−Δ34%**	6.0 mm [5.3-7.0] mm (median [IQR]) **Δ + 2%**	*P* <.0001	CDS: *P* = .004 BWS: *P* <.0001 i-fat: *P* <.0001 LN: *P* = .023 IBUS-SAS: 12 vs. 79 *P* <.0001 −60% vs. 0% *P* <.0001
**Smith^27^**	VEDO (*n* = 8) IFX (*n* = 7) UST (*n* = 6) ADL (*n* = 1) Thiopurine (*n* = 1)	Therapeutic	Week 46	Week 6	*Not reported*	*Not reported*		Δ-16% BWT (AUROC 0.86, *P* = .002, sens 72%, spec 90%) Sonographic response (BWT and CDS) week 6 predictive for treatment response
**Ripollés^28^**	Aminosalycilates (*n* = 9) CS +/- Azathioprine (*n *= 19)	Therapeutic	Week 143	Week 4	*Not reported*	*Not reported*		BWT < 5mm: 25% nonresponder and BWT > 5mm: 67% nonresponder: *P* = ns Hyperaemia grade <2: 18% nonresponse grade ≥2: 76% nonresponse *P* <.01

Intermediate (12-16 weeks) Crohn’s disease								
Author, Year	Treatment	Outcome measure		Time point IUS	Results BWT		*P*-value	Other IUS results
		Type	Time point		Responders	Nonresponders		
**Ripollés^30^**	IFX (*n* = 33) ADL (*n* = 18)	Sonographic remission	Week 52	Week 12	*Not reported*	*Not reported*		Sonographic response or remission at week 12 predicts sonographic response after 1 year (sens 76%, spec 82%; OR 14)
		Sonographic improvement						
**Paredes^29^**	ADL (*n* = 23) IFX (*n* = 13)	Sonographic	Week 52	Week 12	*Not reported*	*Not reported*		All patients with TH at week 12 remained TH at week 52 Patients with TH at week 52 had better long-term outcomes
		Therapeutic	Week 210					
**Lovett^23^**	ADL high dose + thiopurine (*n *= 52) ADL monotherapy (*n* = 25)	Clinical (obstructive symptom score)	Week 52	Week 16	*Not reported*	*Not reported*	ns	
**Saevik^26^**	CS (*n* = 9) ADL (*n* = 1) IFX (*n* = 1) ADL + CS (*n* = 2) IFX + CS (*n* = 1)	Clinical remission and therapeutic	Week 52	Week 12	*Not reported*	*Not reported*	ns	Submucosa lower in responders vs. nonresponders (no data shown, *P* = .04)
**Allocca^16^**	ADL (*n* = 40) IFX (*n* = 8) UST (*n* = 40) VEDO (*n* = 5)	Endoscopic remission	Week 52	Week 12	*Not reported*	*Not reported*		BUS score: 2.89 vs. 5.55 *P* <.01
**Smith^27^**	VEDO (*n* = 8) IFX (*n* = 7) UST (*n* = 6) ADL (*n* = 1) Thiopurine (*n* = 1)	Therapeutic	Week 46	Week 14	*Not reported*	*Not reported*		−Δ10% (AUROC .87, *P* = .001, sens 78%, spec 90%) Sonographic response predicts subsequent treatment response (sens 88%, spec 73%; OR 19)
**Kucharzik^11^**	UST (*n* = 77)	Endoscopic response	Week 48	Week 16	*Not reported*	*Not reported*		−Δ≥25% BWT results in: PPV 27.3 and NPV 75.0
**Ainora^15^**	UST (*n* = 52)	Endoscopic response	Week 48	Week 16	**−Δ20.0%** IQR [−28.6; −16.7]	**−Δ14.3%** IQR [−26.8; 0.0]	ns	CDS: *P* = ns
		Endoscopic remission			**−Δ16.7%** IQR [−28.6; −12.5]	**−Δ16.7%** IQR [−28.6; 0.0]	ns	CDS: *P* = ns

Long-term (>16 weeks) Crohn’s disease								
Author, Year	Treatment	Outcome measure		Time point IUS	Results BWT		*P*-value	Other IUS results
		Type	Timepoint		Responders	Nonresponders		
**Huang^21^**	IFX (*n* = 129)	Endoscopic healing	Week 44-56	14-26 weeks	3.8 mm **−Δ36%**	5.3 mm **−Δ20%**	*P* <.001 *P* <.01	Logistic regression BWS *P* = .019 CDS *P* = .006 i-fat *P* <.001 IBUS-SAS: 23.6 vs. 51.1 (*P* <.001) −34% vs.-20% (*P* <.01)
		**Sonographic transmural healing**			3.6 mm **−Δ39%**	5.2 mm **−Δ20%**	*P* <.001 *P* <.001	Logistic regression BWS *P* <.001 CDS *P* <.001 i-fat *P* <.001 IBUS-SAS: 20.3 vs. 48.7 (*P* <.001) −35% vs. 22% (*P* <.01)
**Lovett^23^**	ADL high dose + thiopurine (*n* = 52) ADL monotherapy (*n* = 25)	Clinical (obstructive symptom score)	Week 52	32 weeks	*Not reported*	*Not reported*	*P* = .003	
**Paredes^29^**	ADL (*n* = 23) IFX (*n* = 13)	Therapeutic	Week 210	52 weeks	*Not reported*	*Not reported*		TH (BWT ≤ 3mm and CDS 0 or 1) was significantly related with a better outcome (OR 11.7, 95% CI: 1.2-108.2; *P* = .01) TH at week 52 resulted in 7.2% events vs. 47% in non-TH group. PPV 92.9%
**Ripollés^30^**	IFX (*n* = 33) ADL (*n* = 18)	Therapeutic	Week 70	52 weeks	*Not reported*	*Not reported*		Patients with sonographic improvement had fewer events than without (89% vs. 35%, *P* <.001; OR 15.48, sens 78% spec 81%) and a lower sonographic score 3 ± 2.3 vs. 5.79 ± 2.33 *P* = .0001
**Dolinger^20^**	IFX (*n* = 14) ADL (*n* = 9) UST (*n* = 21)	Endoscopic remission	Week 52	Week 26	2.0 mm [1.5-2.4] **−Δ53%** [-46 to −66]	5.2 mm [3.9-5.7] ** +Δ7%** [−3 to 11]	*P* <.0001 *P* <.0001	IBUS-SAS: 8 vs. 76 *P* <.0001 Δ-82%vs. -4% *P* <.0001 Responders are more likely to have normalization of all other IUS parameters (values not given)
**Ma^32^**	5-ASA (*n* = 14) MTX/azathioprine (*n* = 33) Anti-TNF (*n* = 14) Thalidomide (*n* = 9 Anti-TNF + thiopurine (*n* = 7)	Steroid-free clinical remission	Week 82	Week 26	*Not reported*	*Not reported*		TH (BWT ≤ 3mm and normal other parameters) had an OR 52.6 *P* <.001
		**Drug escalation**						TH had an OR of 0.1 *P* = .002
		**Hospitalization (CD related)**						TH had an OR of 0.05 *P* = .005
**Ainora^15^**	UST (*n* = 52)	Endoscopic response	Week 48	Week 24	**−Δ33.3%** IQR [−55.0; −20.0]	**−Δ14.3%** IQR [-16.7; 0.0]	*P* <.01	CDS: *P* <.01
		**Endoscopic remission**			**−Δ25.0%** IQR [-33.3; -20.0]	**−Δ16.7%** IQR [-37.5; 0.0]	ns	CDS: *P* = .02

Abbreviations: ADL, adalimumab; Anti*-*TNF, anti-tumor necrotic factor alpha antibody; BUS, bowel ultrasound score; BWS, bowel wall stratification; CD, Crohn’s disease; CDS, color doppler signal; CS, corticosteroids; IBUS-SAS, International Bowel Ultrasound Group-Scoring Activity System; i-fat, inflammatory fat; IFX, infliximab; IQR, interquartile range; IUS, intestinal ultrasound; mLimberg, modified Limberg score; ns, not significant; OR, odds ratio; sc, subcutaneous; SD, standard deviation; Sens, sensitivity; Spec, specificity; TH, transmural healing; UST, ustekinumab; VEDO, vedolizumab. Detailed response definitions for each article are provided in Supplementary Table 1.

Additional IUS parameters were described in 6 studies: 1 out of 5 reported differences in color doppler score (CDS), 2 out of 3 reported differences in inflammatory fat (i-fat), one out of six in bowel wall stratification (BWS).^15^^,^^18^^,^^20^^,^^21^^,^^24^^,^^27^

Four of the studies included three different IUS scores. Two of the studies reported predictive value for the IBUS-SAS (*n* = 2) and the BUS score (*n* = 1), whilst the IUS score used by Cerna et al. was not of significant additional value (Table S2).^16^^,^^17^^,^^20^^,^^21^

#### Rapid IUS (within 4 weeks)

Three studies performed IUS 2 weeks after treatment initiation (Table 1).^18^^,^^22^^,^^27^ Two studies investigating anti-TNF therapy demonstrated a greater reduction in BWT among combined clinical and endoscopic responders, compared to nonresponders at week 12 to 14 (−28% vs. −7% and −3.5% vs. −0.5%, respectively).^18^^,^^22^ One study involving multiple therapies found no significant differences in BWT for therapeutic response at week 46.^27^ Additional IUS parameters were described in one study showing a greater reduction in CDS in combined clinical and endoscopic responders versus nonresponders at week 14.^18^

#### Short-term IUS (4-8 weeks)

Short-term IUS was performed in 10 studies (Table 1).^11^^,^^15^^,^^18–20^^,^^22^^,^^25–28^ In four anti-TNF studies, a greater reduction of BWT at week 4-8 was observed among endoscopic responders compared to nonresponders (ranging from week 10-52).^18^^,^^19^^,^^22^^,^^25^ The reported BWT change varied from −14.6% to −43% vs. +2% to −14% in responders versus nonresponders among studies. One study including either anti-TNF and/or corticosteroids found no significant difference in BWT for therapeutic outcomes at week 52.^26^ However, this study did find a thickened muscularis propria layer as a predictor for future nonresponse.

One of the 2 studies with ustekinumab found that a reduction of less than 25% in BWT at week 8 was predictive for endoscopic nonresponse at week 48 (NPV 80%).^11^ A second study found no difference in BWT change at short-term IUS for endoscopic response at week 48.^15^

Two out of three studies into multiple treatment regiments showed a predictive value of IUS. In one pediatric study, a greater reduction in BWT at week 8 was observed for endoscopic remission after one year (−34% vs. +2%).^20^ A study in adults described that a 16% reduction of BWT had a sensitivity of 72% and specificity 90% to predict treatment success after 46 weeks.^27^ The third study in patients treated with multiple therapies, did not find a significant reduction in BWT at short-term IUS for treatment success at week 143.^28^

Additional IUS parameters were reported in 5 studies.^15^^,^^18–20^^,^^28^ Four out of 5 studies found differences in decrease in CDS, 1 out of 2 in i-fat and 1 out of 2 in BWS. One pediatric study combined parameters and showed a decrease in IBUS-SAS score (−60% vs. 0%).^20^

#### Intermediate IUS (12-16 weeks)

Eight studies performed IUS between week 12 and 16 to assess the subsequent disease course after approximately 1 year ­(Table 1).^11^^,^^15^^,^^16^^,^^23^^,^^26^^,^^27^^,^^29^^,^^30^

Three studies performed IUS following anti-TNF induction. Only one of these studies, including patients with small bowel strictures, assessed the change of BWT alone and did not find a predictive value for clinical response after 1 year.^23^ The other 2 anti-TNF studies combined BWT and CDS. The first study demonstrated that sonographic response, defined as decrease of > 2mm BWT and 1 CDS grade, was a strong predictor of the sonographic outcome (sensitivity 76%, specificity 82%; odds ratio [OR] 14).^30^ Another showed that all patients achieving TH on IUS at week 12 maintained TH on IUS at one year.^29^ In this study, 93% of patients with TH did not need treatment escalation or surgery during long term follow-up (ranged 25-91 months), compared to 47% of patients without TH at week 12 (OR 11.7). Moreover, a study which included either anti-TNF and/or corticosteroids, did not find a significant difference in total BWT for therapeutic outcomes at week 52; however, it did identify a significant difference in the thickness of the submucosal layer.^26^

With ustekinumab treatment, change in BWT after 16 weeks was not predictive for endoscopic response at week 48.^15^ In contrast, a second study with ustekinumab showed that a lack of sonographic response (decrease of <25% BWT) at week 16 was highly predictive for lacking endoscopic response at week 48.^11^ Lastly, a multiple treatment study, demonstrated that achieving sonographic response (BWT −0.5 mm + vascularity improvement) after the induction period was predictive for no need for treatment escalation.^27^

Only one study described CDS separately and found no difference after 16 weeks of ustekinumab treatment.^15^ The BUS score was described in one study, including multiple treatments and did find a difference for endoscopic remission after one year (2.9 vs. 5.6).^16^

#### Long-term IUS (>16 weeks)

Seven studies performed IUS up to 52 weeks after treatment initiation and evaluated long-term outcomes (Table 1).^15^^,^^20^^,^^21^^,^^23^^,^^29^^,^^30^^,^^32^ Intestinal ultrasound had a predictive role in all 4 studies involving anti-TNF treated patients of which only two studies assessed BWT as an individual parameter. A greater decrease of BWT at week 14-26 was demonstrated in endoscopic healers vs. non-healers at week 44-56 (−36% vs. −20%).^21^ Sonographic improvement at 1 year was associated with a lower risk of adverse events in the subsequent year (OR 15).^30^ In addition, one study reported that achievement of TH at one year was predictive of improved therapeutic outcomes and fewer adverse events over the following three years (OR 12).^29^ In the fourth study, BWT after 32 weeks was predictive for improvement of obstructive complains after 1 year in patients with small bowel strictures.^23^

Across two studies including multiple therapies, a reduction in BWT at week 26 was predictive of endoscopic remission after 19 months (−53% vs. +7%) in one study, whereas in the other, a decrease in BWT was associated with longer time to drug escalation or reduced hospitalization rates, and a higher probability of steroid-free clinical remission.^20^^,^^32^ In ustekinumab treated patients, IUS after 24 weeks showed a greater BWT decrease for endoscopic response at week 48 (−33.3% vs. −14.3%), but not for endoscopic remission.^15^

Two studies described other individual IUS parameters for endoscopic outcomes. One study described a difference for CDS, i-fat, and BWS separately at week 14-26.^21^ Another study also showed a CDS difference at week 24 for endoscopic response at week 48.^15^

Two studies reported a difference at IBUS-SAS at week 14-26 and >16 weeks, respectively (24 vs. 51 and 8 vs. 76).^20^^,^^21^

#### Pooled data analysis in anti-TNF treatment for CD

The largest subgroup in this SR based on treatment type was anti-TNF (total *n* = 649). Seven out of 14 authors provided BWT measurements for a total of 236 patients with clinical (*n* = 51), therapeutic (*n* = 38), and endoscopic (*n* = 147) outcomes (Table 2).^18–20^^,^^22^^,^^24^^,^^27^^,^^30^ Baseline IUS could not predict future response.

**Table 2.** Pooled data analysis of mean change in bowel wall thickness (%) categorized per outcome in Crohn’s disease patients treated with anti-tumor necrosis factor (TNF).

		All outcomes mean % ΔBWT (*n*)	Clinical mean % ΔBWT (*n*)	Therapeutic mean % ΔBWT (*n*)	Endoscopic mean % ΔBWT (*n*)
**Week 4-8**	Responder	−27% (*n* = 95)		−20% (*n* = 4)	−27% (*n* = 91)
	Nonresponder	−5% (*n* = 46)		−1% (*n* = 2)	−5% (*n* = 44)
	*P*-value	*P* <.001		*P*=.234	*P*<.001
**Week 12-16**	Responder	−23% (*n*=123)	−19% (*n*=37)	−18% (*n*=28)	−29% (*n*=58)
	Nonresponder	−4% (*n*=48)	−10% (*n*=14)	+2% (*n*=8)	−2% (*n*=26)
	*P*-value	*P*<.001	*P*=.263	*P*=.011	*P*<.001

Abbreviation: BWT,  bowel wall thickness.

**Figure 2.**
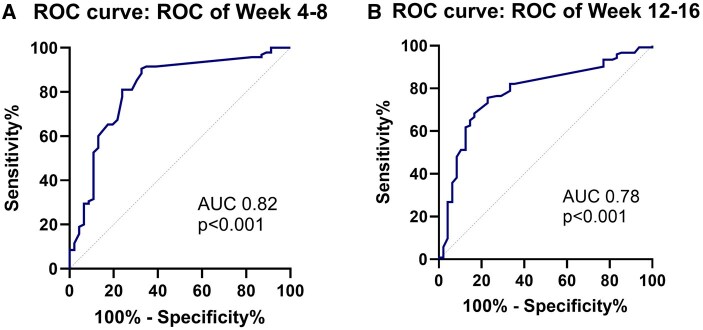
Percentage change in bowel wall thickness from baseline at week 4-8 (A) and week 12-16 (B) to predict treatment response based on pooled data from anti-TNF treatment in CD patients. AUC, Area under the ROC curve.

Intestinal ultrasound performed after 4-8 weeks (*n* = 141) showed a greater reduction in BWT in responders compared to nonresponders (ranging between 12 and 52 weeks).^18–20^^,^^22^^,^^27^ The AUROC for BWT was 0.82 (95% confidence interval [CI], 0.73-0.90) (Figure 2A). A cutoff value of ≥25% decrease in BWT corresponded to 89% specificity (95% CI 80%-98%) and a sensitivity of 52% (95% CI 42%-62%) for treatment success with a PPV of 91% (95% CI 83%-98%), NPV of 47% [95% CI 37%-58%] and OR of 8.7 (3.2-24.0). Using the Youden index, the optimal cutoff was a decrease of ≥12.5% BWT providing a specificity of 76% (95% CI 64-88%]) and sensitivity of 81% (95% CI 73%-89%).

After 12-16 weeks (*n* = 171), there was a greater decrease in BWT between responders compared to nonresponders.^18^^,^^20^^,^^22^^,^^24^^,^^27^^,^^30^ The AUROC for BWT was 0.78 (95% CI 0.71-0.86) (Figure 2B).

A cutoff value of ≥25% decrease in BWT corresponded to 88% specificity (95% CI 78%-97%) and a sensitivity of 55% (95% CI 47%-64%) for treatment success with a PPV of 92% (95% CI 86%-98%), NPV of 43% (95% CI 33%-53%), and OR of 8.7 (95% CI, 3.4-21.9). Using the Youden index, the optimal cutoff was a decrease of ≥20% BWT providing a specificity of 83% (95% CI, 73%-94%) and sensitivity of 67% (95% CI 58%-75%).

For mean differences per outcome see Table 2. When differentiating per outcome type at week 12-16, endoscopic outcomes showed the highest area under the ROC (Figure S1, see online supplementary material for a color version of this figure).

### Prognostic value of IUS in UC

#### Baseline IUS (before treatment start)

The predictive value of baseline IUS characteristics in UC was described in eight studies (Table 3).^6^^,^^33–39^ In total 6 studies assessed BWT as an individual parameter. Five studies found no significant difference in BWT between endoscopic and/or clinical responders and nonresponders at ranging from week 8 to 26.^6^^,^^33–36^^,^^38^ A study in children with UC did find a difference in pretreatment BWT for endoscopic response after 6-12 months (BWT 4.3 mm vs. 5.6 mm), but not for endoscopic remission.^37^

**Table 3.** Overview of current available studies assessing the predictive value of intestinal ultrasound for treatment response in ulcerative colitis.

Baseline ulcerative colitis							
Author, Year	Treatment	Outcome measure		Results BWT		*P*-value	Other IUS results
		Type	Time point	Responders	Nonresponders		
**Parente^35^**	CS (*n* = 74)	Endoscopic remission	Week 65	*Not reported*	*Not reported*		Ultrasound score 2 vs. 3: OR 7.1
**Sagami^36^**	CS (*n* = 36) Anti-TNF (*n* = 23) VEDO (*n* = 20) TOFA (*n* = 9) UST (*n* = 6) Tacrolimus (*n* = 6)	Clinical remission	Week 8	Ascending 2.4 ± 1.6 mm (mean ± standard deviation [SD])	Ascending 2.5 ± 1.8 mm (mean ± SD)	ns	CDS *P* = ns
				**Transverse 3.0 ± 1.8 mm**	Transverse 2.8 ± 1.7 mm		
				**Descending 4.0 ± 1.8 mm**	Descending 3.9 ± 1.6 mm		
				**Sigmoid colon 4.0 ± 1.6 mm**	Sigmoid colon 4.3 ± 1.3 mm		
				**Rectum 7.0 ± 1.6 mm**	Rectum 6.8 ± 1.6 mm		
**De Voogd^6^**	Tofa (*n* = 27)	Endoscopic response	Week 8	*Not reported*	*Not reported*	ns	
		**Endoscopic remission**					
**De Voogd^33^**	IFX (*n* = 18) ADL (*n* = 1) VEDO (*n* = 14) UST (*n* = 2) TOFA (*n* = 14) Ciclosporin (*n* = 1)	Endoscopic improvement	Week 8-26	4.8 ± 1.59 mm (mean ± SD)	5.2 ± 1.04 mm (mean ± SD)	ns	
		**Endoscopic remission**		4.9 ± 1.77 mm	5.0 ± 1.12 mm		
**Ollech^34^**	TOFA (*n* = 27)	Clinical response	Week 8	4 [3.3-4.3] mm (median [interquartile range, IQR])	4.4 [3.85-4.9] mm (median [IQR])	ns	CDS *P* = ns MUC: *P* = ns
**El-Nady^38^**	Anti-TNF (UC *n* = 33, CD *n* = 12)	According to ECCO guidelines	Week 12	5.7 ± 1.55 mm (mean ± SD)	5.5 ± 1.69 mm (mean ± SD)	ns	
**Dolinger^37^**	IFX (*n* = 12) ADL (*n* = 1) USTE (*n* = 11) VEDO (*n* = 8) TOFA (*n* = 5) UPA (*n* = 3) Ozanimod (*n* = 2)	Endoscopic improvement	Week 26-52	4.2 [3.8-5.5] mm (median [IQR])	5.6 [4.8-6.0] mm (median [IQR])	*P* = .02	
		**Endoscopic response**		4.3 [3.8-5.2] mm	5.6 [5.2-6.0] mm	*P* = .01	
		**Endoscopic remission**		4.1 [3.8-5.6] mm	5.0 [4.6-5.6] mm	ns	IBUS-SAS 37.0 vs. 63.8 *P* = ns MUC 7.7 vs. 9.0 *P* = ns Civitelli index 2 vs. 2 *P* = ns
**Allocca^39^**	IFX (*n* = 29) ADL (*n* = 4) VEDO (*n* = 14) UST (*n* = 2)	Endoscopic response	Week 41	*Not reported*	*Not reported*		MUC *P* = .03 *Values not reported*

Rapid (<4 weeks) ulcerative colitis								
Author, Year	Treatment	Outcome measure		IUS time point	Results BWT		*P*-value	Other IUS results
		Type	Timepoint		Responders	Nonresponders		
**Sagami^36^**	CS (*n* = 36) Anti-TNF (*n* = 23) VEDO (*n* = 20) TOFA (*n* = 9) UST (*n* = 6) Tacrolimus (*n* = 6)	Clinical	Week 8	Week 1	*Not reported*	*Not reported*	*P* <.05	CDS (values not reported) *P* <.05 Odds ratio (OR) 1.9 for BWT decrease of 1 mm
**De Voogd^33^**	IFX (*n* = 18) ADL (*n* = 1) VEDO (*n* = 14) UST (*n* = 2) TOFA (*n* = 14) Ciclosporin (*n* = 1)	Endoscopic improvement	Week 8-26	Week 2	3.25 ± 1.40 mm (mean ± SD) **−Δ32%**	4.13 ± 1.20 mm (mean ± SD) **−Δ20%**	ns	CDS, BWS, haustration and i-fat: ns ΔLN *P* = .03
		**Endoscopic remission**			3.28 ± 1.61 mm **−Δ34%**	3.89 ± 1.22 mm **−Δ24%**		
**Yoshida^40^**	Cytapheresis (*n* = 26) In addition, CS, 5-asa and immunomodulator	Clinical remission	Week 52	Week 2-3	*Not reported*	*Not reported*	*P* <.05	90.9% of patients who had a decrement of 2.5 mm after 2-3 weeks treatment maintained clinical remission at 1 year compared to 40.0% in the nonresponse group *P* <.05

Short-term (4-8 weeks) ulcerative colitis								
Author, Year	Treatment	Outcome measure		IUS time point	Results BWT		*P*-value	Other IUS results
		Type	Timepoint		Responders	Nonresponders		
**De Voogd^33^**	IFX (*n* = 18) ADL (*n* = 1) VEDO (*n* = 14) UST (*n* = 2) TOFA (*n* = 14) Ciclosporin (*n* = 1)	Endoscopic improvement	Week 8-26	Week 6	3.01 ± 1.21 mm (mean ± SD) −Δ37%	4.12 ± 1.26 mm (mean ± SD) −Δ20%	*P* = .002	CDS and BWS *P* = ns Loss of haustration, i-fat or LN *P* <.05
		**Endoscopic response**			−Δ28%	−Δ9%	ns	
		**Endoscopic remission**			2.51 ± 1.18 mm −Δ49%	4.07 ± 1.11 mm −Δ19%	*P* = .026	
**Dolinger^37^**	IFX (*n* = 12) ADL (*n* = 1) USTE (*n* = 11) VEDO (*n* = 8) TOFA (*n* = 5) UPA (*n* = 3) Ozanimod (*n* = 2)	Endoscopic improvement	Week 26-52	Week 8	2.0 [1.7-3.0] mm −Δ52%	4.3 [3.4-4.7] mm −Δ23%	*P* = .002	
		**Endoscopic response**			2.1 [1.7-3.1] mm (median [IQR]) −Δ51%	4.1 [3.1-4.9] mm (median [IQR]) −Δ27%	*P* = .01	
		**Endoscopic remission**			1.8 [1.6-2.7] mm −Δ55%	3.5 [2.4-4.4] mm −Δ38%	*P* = .003 *P* = .02	IBUS-SAS 13.4 vs. 31.6 *P* = .01 MUC 4.2 vs. 6.2 *P* = .01 Civitelli index 1 vs. 2 *P* = .04

Intermediate (12-16 weeks) ulcerative colitis								
Author, Year	Treatment	Outcome measure		IUS time point	Results BWT		*P*-value	Other IUS results
		Type	Time point		Responders	Nonresponders		
**Allocca^39^**	IFX (n = 29) ADL (*n* = 4) VEDO (*n* = 14) UST (*n* = 2)	Endoscopic response	Week 41	Week 12	*Not reported*	*Not reported*		MUC ≤6.2 OR 5.8 *P* = .010
		**Endoscopic remission**						MUC ≤4.3 OR 10.4 *P* = .041
**Parente^35^**	CS (*n* = 74)	Endoscopic remission	Week 65	Week 12	*Not reported*	*Not reported*		Ultrasound score ≥2 has a high risk of severe endoscopic activity (OR: 9.1)

Long-term (>16 weeks) ulcerative colitis								
Author, Year	Treatment	Outcome measure		IUS time point		Results BWT	*P*-value	Other IUS results
		Type	Time point		Responders	Nonresponders		
**Parente^35^**	CS (*n* = 74)	Endoscopic remission	Week 65	Week 39	*Not reported*	*Not reported*		Ultrasound score ≥2 has a high risk for severe endoscopic activity (OR 14.8)

Abbreviations: Anti*-*TNF, anti-tumor necrotic factor alpha antibody (infliximab, adalimumab, golimumab); BWS, bowel wall stratification; CDS, color doppler signal; CI, confidence interval; CS, corticosteroids; i-fat, inflammatory fat; IQR, interquartile range; IUS, intestinal ultrasound; MUC, Milan Ultrasound Criteria; ns, not significant; OR, odds ratio; SD, standard deviation; Sens, sensitivity; Spec, specificity; TH, transmural healing; TOFA, tofacitinib; UST, ustekinumab; VEDO, vedolizumab.  Detailed response definitions for each article are provided in Supplementary Table 1.

Two studies describing CDS found no difference at baseline between responders and nonresponders.^34^^,^^36^ Four studies evaluated IUS scores at baseline. The Milan Ultrasound Criteria (MUC) were assessed in 3 studies, with 1 study showing a difference.^34^^,^^37^^,^^39^ The IBUS-SAS and the Civitelli index were each evaluated in a single pediatric study, neither showing a difference.^37^ In addition, one study in patients treated with prednisolone reported an OR of 7.1 for the presence of severe inflammation on endoscopy after 15 months, when having an U.S. score of 3 (BWT ≥ 8mm with increased CDS) compared to an U.S. score of 2 (BWT 6-8 mm with increased CDS) at baseline (Table S2).^35^

#### Rapid IUS (within 4 weeks)

Three studies assessed rapid IUS response (Table 3).^33^^,^^36^^,^^40^ One study including multiple therapies, reported that a decrease of 1 mm BWT in the rectum, assessed by perineal ultrasound at 1 week, could predict clinical response at week 8 (OR 1.9).^36^ Another study with multiple treatments found no overall difference in early reduction of BWT for endoscopic response and nonresponse at week 8-26.^33^ A study with cytapheresis found that those with a decrease in BWT of 2.5 mm at week 2 had a higher rate of clinical remission (91% vs. 40%) after one year.^40^

Two studies described CDS as individual IUS parameter with conflicting results.^33^^,^^36^ One of these studies also mentioned other IUS parameters and described no change from baseline in i-fat, BWS and haustration, while presence of lymph nodes decreased in responders.^33^

#### Short-term IUS (4-8 weeks)

Two studies described short-term IUS, both including different types of treatment using endoscopic outcomes (Table 3).^33^^,^^37^ At week 6, a difference in BWT was seen for endoscopic improvement and remission at week 8-26 (BWT −37% vs. −20% and −49% vs. −19%), but not for response.^33^ However, in a pediatric study, a difference in BWT for endoscopic improvement, response, and remission at week 26-52, respectively (BWT −52% vs. 23%; −51% vs. 27% and −55% vs. −38%) was reported.^37^

One study described other IUS parameters separately, there was a difference in loss of haustration, i-fat, and presence of lymph nodes between responders and nonresponders, whereas no differences were observed in CDS and BWS.^33^ In children 3 IUS scores-including MUC, IBUS-SAS, Civitelli index-showed differences in absolute scores, absolute change in scores and % change.^37^

#### Intermediate IUS (12-16 weeks)

Two studies were identified, one analysing prednisolone and another multiple biologicals, both using ultrasound scores incorporating both BWT and CDS and performed IUS at week 12 with a long-term endoscopic outcome (week 41-65) (Table 3).^35^^,^^39^

The study with prednisolone treatment found that an ultrasound score of ≥2 (BWT > 6mm and presence of CDS) had an OR of 9.12 for moderate-severe endoscopic Baron score at 16 months.^35^ The study with multiple biologicals found that a MUC ≤6.2 had an OR of 5.8 for endoscopic response and MUC ≤4.2 for endoscopic remission at 10 months with an OR of 10.4.^39^

#### Long-term IUS (>16 weeks)

The predictive value of late IUS is described in one study looking into prednisolone treatment (Table 3).^35^ Having an ultrasound score of ≥2 (BWT > 6mm and presence of CDS) after 9 months was predictive (OR 14.8) for a moderate-severe endoscopic Baron score at 15 months.

### Prognostic value of IUS in acute severe UC

Four articles were included in the analysis of ASUC. Two of these originated from the same patient cohort, with one being the long-term follow-up of the other (Table 4).^31^^,^^41–43^

**Table 4.** Overview of current available studies assessing the predictive value of intestinal ultrasound for treatment response in acute severe ulcerative colitis.

ASUC								
Author, Year	Treatment	Outcome measure		IUS time point	Results BWT		*P*-value	Other IUS results
		Type	Time point		Responders	Nonresponders		
**Smith^31^**	CS IV (*n* = 10)	Therapeutic	Within 7 days	<24 h	Median 4.6 mm (range 4.2-5.6)	Median 6.2 mm (range 6-7.9)	*P* = .009	CDS and BWS: *P* = ns
			**Day 7**	Day 3	Median 4.0 IQR mm [3.5-4.5] **−Δ13%**	Median 6.3 mm [5.5-6.9] ** +Δ2%**	*P* = .009	
			**Week 13**	Week 2	Median 2.6 mm (range 1.8-4.7) **−Δ43%**	Median 5.2 mm (*n* = 1) **−Δ16%**	*Not reported*	
**Ilvemark^41^**	CS iv (n = 56)	Clinical	Day 7	Day 0	Median 4.9 mm interquartile range (IQR) (4.2-5.6)	Median 5.0 mm IQR (4.6-6.5)	ns	CDS, BWS, i-fat and haustration: *P* = ns
				**Day 2**	Median 3.1 mm IQR [2.5-4.0] **−Δ35.9%**	Median 4.9 mm IQR [4.2-5.2] **−Δ4.1%**	*P* = .000016	CDS: *P* = ns BWS *P* = .04 i-fat *P* = .003 Haustration *P* = .01
				**Day 6**	Median 2.5 mm IQR [2.5-4.0] **−Δ54.9%**	Median 4.9 mm IQR [4.2-5.2] **−Δ8.2%**	*P* = .000018	CDS, BWS and i-fat: *P* = ns Haustration *P* = .026
		**Therapeutic**		Day 0	Median 4.9 mm IQR [4.2-5.9]	Median 5.0 mm IQR [4.4-6.8]	ns	CDS, BWS and i-fat: *P* = ns Haustration *P* = .014
				**Day 2**	Median 3.1 mm IQR [2.7-4.3] **−Δ34.0%**	Median 4.4 mm IQR [3.9-5.5] **−Δ9.6%**	*P* = .0023	CDS *P* = .04 BWS and i-fat: *P* = ns Haustration *P* = .01
				**Day 6**	Median 2.7 mm IQR [2.0-3.8] **−Δ47.3%**	Median 4.2 mm IQR [2.9-5.9] **−Δ13.0%**	ns	CDS, BWS and i-fat and haustration: *P* = ns
**Ilvemark^43^**	CS IV (*n* = 56)	Therapeutic	Within 13 weeks	Day 0	Median 4.9 mm IQR [4.3-6.0]	Median 5.0 mm IQR [4.5-5.9]	ns	
				**Day 2**	Median 3.6 mm IQR [2.7-4.7] −**Δ28.2%**	Median 4.8 mm IQR [4.3-5.3] **−Δ5.5%**	*P* = .03 *P* = .04	Multivariate analysis: CDS, BWS, i-fat and haustration: *P* = ns
				**Day 6**	Median 2.9 mm IQR [2.1-3.9] **−Δ-3.0%**	Median 5.4 mm IQR [4.2-5.7] **−Δ0.6%**	*P* = .02 *P* = .02	Multivariate analysis: CDS, BWS and haustration: *P* = ns i-fat *P* = .02
			**Within 52 weeks**	Day 0	Median 4.9 mm IQR [4.4-6.1]	Median 4.9 mm IQR [4.2-5.9]	ns	
				**Day 2**	Median 3.6 mm IQR [2.7-4.7] **−Δ28.6%**	Median 4.4 mm IQR [4.2-5.0] **−Δ5.5%**	ns *P* = .03	Multivariate analysis: CDS, BWS, i-fat and haustration: *P* = ns
				**Day 6**	Median 2.9 mm IQR [2.1-4.1] **−Δ43.2%**	Median 4.2 mm IQR [3.5-5.4] **−Δ0.6%**	ns *P* = .02	Multivariate analysis: CDS, BWS and haustration: *P* = ns i-fat *P* = .02
				**Week 13**	Median 3.0 mm IQR [2.2-3.7] **−Δ46.4%**	Median 4.6 mm IQR [4.2-5.1] **−15.3%**	ns	
**Yamagucy^42^**	Cytapheresis (*n* = 26), concomitant: sulfasalazine/ mesalamine (*n* = 21) Azathioprine (*n* = 6) CS (*n* = 16)	Clinical	Day 7	Day 0-5	Mean 5.1 ± 1.5 mm	Mean 5.1 ± 1.5 mm	ns	CDS and BWS: *P* = ns

Abbreviations: BWS, bowel wall stratification; CDS, color doppler signal; CS, corticosteroids; h, hour; i-fat, inflammatory fat; IQR, interquartile range; IUS, intestinal ultrasound; IV, intravenous; ns, not significant.  Detailed response definitions for each article are provided in Supplementary Table 1.

Only the study with short and long-term follow up performed all IUS prior to the start of IV corticosteroid treatment. For short-term outcomes pretreatment IUS was unable to predict clinical remission or the need for salvage therapy at day 7.^41^^,^^43^ After 48 hours of intravenous corticosteroid treatment a ≤20% BWT reduction predicted clinical nonresponse (AUROC 0.85, sensitivity 84.2%, specificity 78.4%) and need for salvage therapy at day 7 (AUROC 0.74, sensitivity 73.7%, and specificity 73.0%). The long-term follow-up of this cohort described the need for colectomy within 3 and 12 months. Baseline BWT could not predict the need for colectomy within 1 year, but none of the patients who had a BWT < 3mm after 48 hours went through colectomy during the 1-year follow-up (*P* = 0.04). Moreover, a BWT > 4mm after 48 hours was a significant predictor for colectomy at 3- and 12-months follow-up (OR 9.5; OR 5.6, respectively). A BWT >4 mm on day 6 remained predictive for colectomy.

The other 2 studies performed the first IUS after starting treatment. One study in prednisolone treated patients performed an IUS within the first 24 hours and after 3 days. At both IUS time points BWT could predict need for salvage therapy within 7 days (at 24 h: BWT 4.6 vs. 6.2 mm and at 3 days: 4.0 vs. 6.3 mm).^31^ Notably, this study stated that a patient with any colonic segment with a BWT > 6mm needed infliximab salvage therapy. For cytapheresis, IUS performed within the first 5 days was not able to predict clinical outcomes within the first week.^42^

None of the 4 studies in ASUC described differences in CDS and BWS at the first IUS time point and results are inconsistent at later time points (Table 4). Intestinal ultrasound scores were not described in the included ASUC studies.

### Risk of bias assessment

The risk of bias assessment for the 31 included studies is presented in (Table 5). In the domain of study participation, 3 studies were rated as high risk and 8 as moderate, primarily due to poorly defined inclusion and exclusion criteria. For study attrition, 2 studies were rated high risk and 10 moderate risk, owing to substantial participant dropout without clearly reported reasons. In the prognostic factor measurement domain, 3 studies had high and 7 moderate risk of bias, largely due to inadequate description of methods used to assess IUS parameters. The highest risk of bias was observed in the study confounding domain, with 7 studies rated high and 17 moderate, often due to non-generalizable populations (e.g., only biologic-naïve or refractory patients) or the inclusion of multiple treatments without appropriate subgroup analyses. Only one study was rated as having moderate risk in the statistical analysis domain, due to insufficient detail in the description of statistical methods.

**Table 5.** Quality assessment included studies.

Study	IBD type	Study participation	Study attrition	Prognostic factor measurement	Outcome measurement	Study confounding	Statistical analysis and reporting
**Ainora^15^**	CD	Low	Moderate	Moderate	Low	Moderate	Low
**Allocca^16^**	CD	Low	Low	Low	Low	Moderate	Low
**Cerna^17^**	CD	High	Low	High	Moderate	High	Low
**Chen^18^**	CD	Low	Moderate	Low	Low	Moderate	Low
**Dolinger^20^**	CD	Low	Low	Low	Low	Moderate	Low
**Huang^21^**	CD	Low	Moderate	Low	Moderate	Moderate	Low
**Kucharzik^11^**	CD	Moderate	Moderate	Low	Low	Low	Low
**Laterza^22^**	CD	Low	Moderate	Low	Low	Moderate	Low
**Lovett^23^**	CD	Moderate	High	Low	Moderate	Moderate	Low
**Ma^32^**	CD	Low	Moderate	Low	Low	High	Low
**Orlando^24^**	CD	Low	Low	Low	Low	Low	Low
**Paredes^29^**	CD	Low	Low	Moderate	Low	Low	Low
**Quaia^25^**	CD	Low	Moderate	Moderate	Moderate	High	Low
**Ripollés^28^**	CD	High	Low	Moderate	Low	High	Low
**Ripollés^30^**	CD	Low	Low	Moderate	Moderate	Low	Low
**Saevik^26^**	CD	Moderate	Moderate	Low	Moderate	High	Low
**Smith^27^**	CD	Low	Moderate	Low	Low	Moderate	Low
**de Voogd^19^**	CD	Low	Low	Low	Low	Moderate	Low
**Allocca^39^**	UC	Low	Low	Low	Low	Moderate	Low
**Dolinger^37^**	UC	Low	Low	Low	Low	Moderate	Low
**Ollech^34^**	UC	Low	Low	Moderate	Moderate	Low	Low
**Parente^35^**	UC	Low	High	Low	Low	Moderate	Low
**Sagami^36^**	UC	Low	Low	Low	Low	Moderate	Low
**De Voogd^6^**	UC	Low	Low	Low	Low	Moderate	Low
**De Voogd^33^**	UC	Moderate	Moderate	Low	Low	Moderate	Low
**Yoshida^40^**	UC	High	Low	Moderate	Moderate	High	Moderate
**El-Nady^38^**	IBD	Moderate	Low	High	Moderate	Moderate	Low
**Ilvemark^41^**	ASUC	Moderate	Low	Low	Low	Low	Low
**Ilvemark^43^**	ASUC	Low	Low	Low	Low	Moderate	Low
**Smith^31^**	ASUC	Moderate	Low	Low	Low	Low	Low
**Yamagucy^42^**	ASUC	Moderate	Low	High	Moderate	High	Low

Abbreviations: ASUC, acute severe colitis; CD, Crohn’s disease; IBD, inflammatory bowel disease; UC, ulcerative colitis.

### Proposal for clinical implementation

**Figure 3.**
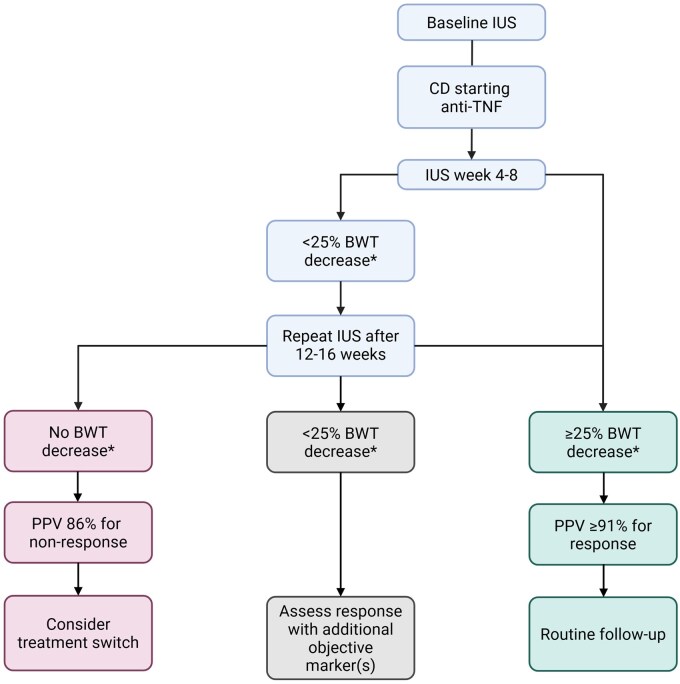
Proposed flowchart for IUS monitoring in CD patients starting on anti-tumor necrosis factor (TNF) therapy. CD = Crohn’s disease, IUS = intestinal ultrasound, BWT = bowel wall thickness. ^a^BWT decrease compared to baseline IUS.

In Figure 3, we propose an IUS algorithm for CD patients starting anti-TNF, based on the results our pooled data analysis.

## 4. Discussion

This systematic review provides a comprehensive overview of the predictive role of IUS following treatment initiation in IBD, showing that IUS can predict response already at an early stage after treatment initiation, both in CD as well as in UC.

In CD, only a limited number of studies have assessed the predictive value of IUS within the first 4 weeks of treatment initiation. Significant differences in BWT between responders and nonresponders were reported in 2 studies evaluating anti-TNF therapy with endoscopic outcomes. However, only one of these studies reported clinically meaningful differences. From week 4-8 onward, the majority of studies report significant differences in IUS parameters—particularly in BWT—between responders and nonresponders. At later time points, an increasing number of studies report significant differences between responders and nonresponders, based on absolute BWT values, BWT reduction, or composite IUS scores. In UC, fewer studies are available, but a similar pattern emerges. Two out of 3 studies reported differences in BWT change between responders and nonresponders within the first 4 weeks. However, the clinical applicability of these studies is limited as one evaluated a therapy not aligned with current guidelines and the other utilized transperineal ultrasound. Notably, a recently published post hoc analysis of this transperineal ultrasound study demonstrated that a reduction in rectal BWT after 1 week not only predicted short-term symptomatic improvement but also subsequent endoscopic outcomes. Although only 2 studies performed IUS between 4 and 8 weeks in UC, both studies showed a clear difference between responders and nonresponders. In ASUC, the absolute BWT and the change in BWT after starting IV steroid treatment from 1-3 days onward is predictive for the need for rescue therapy or colectomy. Accordingly, 1 small study in only 10 patients showed a difference in BWT after 24 hours suggesting that IUS could already be predictive at this time point.

Current literature on IUS scores has well established the utility for distinguishing between active and inactive disease. In the more recent studies included in this review, multiple IUS parameters or composite scores were reported more frequently. However, data on the responsiveness of these scores remain limited. For both CD and UC, only four studies have evaluated the predictive role of IUS indices at different time points, of which only two studies (one in pediatric UC and one in pediatric CD) assessed an early time point. Our pooled data analysis represents the largest cohort (*n* >200) examining the predictive value of IUS for response to anti-TNF treatment. The study population included in this pooled data analysis is representative of the general CD population with a wide range of disease duration (from several months up to 10 years) and included both biologic naïve and exposed patients. For the pooled data analysis, we aimed for a high specificity of 90% as in clinical practice it is most relevant to detect nonresponse as early as possible, while at the same time minimizing inappropriate treatment changes in patients with a delayed response. This specificity was reached at BWT decrease of ≥25% at week 4-8 and 12-16, and was highly predictive of treatment response (PPV 91% and PPV 92%, respectively). This reduction in BWT aligns with a recent Delphi expert-consensus statement suggesting a 25% reduction in BWT as sonographic response.^44^ Lacking a BWT decrease after anti-TNF induction is highly predictive for nonresponse (PPV 86%). In patients with an intermediate BWT reduction (<25%), prediction of treatment response remains uncertain. In this group, additional objective markers (e.g., fecal calprotectin) may help to improve classification. Future studies are needed to evaluate the added value of such markers and to determine whether they can increase the sensitivity of early response detection. Accordingly, we propose a flowchart for use of IUS in clinical practice for CD patients treated with anti-TNF (Figure 3).

There are several factors that influence the kinetics of response on IUS, such as the treatment modality, disease location, disease duration, and/or previous exposure to advanced treatments. In UC, a drug-specific time kinetic effect was observed at 2 weeks after treatment initiation for anti-TNF and JAK-inhibitors, which appeared faster than Vedolizumab. By week 6 this difference disappeared.^33^ In line with this, another UC study demonstrated a significant decrease in BWT as early as 1 week after treatment initiation in a cohort predominately treated with anti-TNF, JAK-inhibitors, and/or corticosteroids.^36^ In CD, data on differences in treatment kinetics are lacking, although several studies have shown that anti-TNF therapy can already induce measurable differences in BWT between responders and nonresponders within 2 weeks.^18^^,^^22^ These studies show that it is necessary to establish the optimal time point to assess ultrasound response per treatment class. With the increasing incorporation of ultrasound in clinical trials, data on the kinetics of ultrasound response per treatment class are likely to become available in the near future. Studies show that biologic naïve patients have a more pronounced IUS response to treatment compared to bio-exposed patients.^11^ Disease type and location also influence treatment outcomes: BWT decreases slower in the terminal ileum than in the colon and changes occur swifter in UC than in CD when treated with the same treatment class.^11^^,^^15^^,^^44^

There are several strengths of this manuscript. To our knowledge this is the first systematic review to evaluate the (early) predictive value of IUS for treatment response in IBD. The importance of IUS is increasingly recognized, as indicated by its adaptation into the ECCO-ESGAR-ESP-IBUS guideline on Diagnostics & Monitoring IBD.^12^ Therefore, a summary of its predictive value for treatment response is a timely topic. In addition to this guideline, this systematic literature review provides a systematic analysis of individual and combined IUS parameters and their change after starting treatment at different time points. Furthermore, we provide an algorithm for clinical practice based on the pooled data analysis with access to raw BWT measurements on anti-TNF-treated CD patients. Finally, the focus on early prediction highlights IUS as a valuable monitoring tool.

Several limitations should be acknowledged. Substantial heterogeneity was observed across studies. The timing of IUS differed per study, which we addressed by categorizing the time points. All studies reported BWT, while only nine studies reported CDS, and even fewer reported BWS and i-fat. Therefore, we focused on BWT, which is considered the most reliable and reproducible IUS parameter.^44^ Furthermore, we were unable to correct for treatment, disease location, and duration, which are relevant in IUS kinetics as previously described. Moreover, the outcome definition differed per study (Table S1). We tried to overcome this by categorizing the outcomes into several broad categories. The importance of the differences in these outcomes is underlined by the results from our pooled data analysis, which showed different changes in BWT per treatment outcome. More specifically, BWT was more strongly associated with endoscopic outcomes than with clinical outcomes (Figure S1, see online supplementary material for a color version of this figure).

Large multi-center studies are needed to accurately determine the optimal thresholds and time points of IUS to predict response in CD and UC. These studies should include multiple therapies and analyze outcomes separately by type of treatment, use predefined time points, take disease location into account and clearly defined uniform outcomes. Additionally, incorporating standardized IUS parameters and implementation of IUS scores may improve the predictive value.

## 5. Conclusion

Intestinal ultrasound predicts response both in CD and UC already in the early stages of treatment. In anti-TNF treated CD patients decrease in BWT equal or greater than 25% after 4-8 weeks and 12-16 weeks accurately predicts treatment response.

## Supplementary Material

jjag017_Supplementary_Data

## Data Availability

Data, analytic methods, and study materials will be made available to other researchers on reasonable request.
