# Supplementary material for: The predictive value of intestinal ultrasound for treatment response in inflammatory bowel disease: a systematic review and pooled data analysis
Source: J Crohns Colitis. 2026 Apr 15;20(4):jjag017. doi: 10.1093/ecco-jcc/jjag017 (PMC13080699; doi:10.1093/ecco-jcc/jjag017)
Supplement: jjag017_Supplementary_Data [file jjag017_supplementary_data.zip › Supplementary Material 2. Risk of Bias Assessment QUIPS Tool.docx]

**Supplementary Material 2. Risk of Bias Assessment QUIPS Tool**

Predefined QUIPS Criteria

**Study Participation**

- **Low risk of bias**: Patients starting with remission induction therapy, description of time period, location, clear in- and exclusion criteria and a baseline table
- **Moderate risk of bias:** One of the above missing
- **High risk of bias:** Two or more of the above missing

**Study Attrition**

- **Low risk of bias**: No loss to follow-up or adequate description and imputation of missing data.
- **Moderate risk of bias**: Few patients lost to follow-up or inadequate description/imputation of missing data or suspected non-reporting of loss to follow-up.
- **High risk of bias:** Many patients lost to follow-up without adequate description/imputation of missing data.

**Prognostic Factor Measurement**

- **Low risk of bias:** Defined cut-off for bowel wall thickness (BWT), clear description of BWT measurements, BWT measurements in cross-sectional and longitudinal plane, measurements at follow up time points in same affected segment, clear description of other IUS parameters, maximum of 20% missing IUS.
- **Moderate risk of bias:** Definition missing one of the aspects mentioned above.
- **High risk of bias:** Definition of prognostic factor absent or missing more than one of the aspects mentioned.

**Outcome Measurement**

- **Low risk of bias**: Clearly defined reference standard, blinded, all participants received same reference standard
- **Moderate risk of bias:** No clearly defined reference standard or not blinded
- **High risk of bias:** Different reference standards and not blinded

**Study Confounding**

- **Low risk of bias:** One treatment included, with specified dose either mentioned in article or by protocol, in a generalized population
- **Moderate risk of bias:** One of the above not specified
- **High risk of bias:** More than one of the above not specified

**Statistical Analysis and Reporting**

- **Low risk of bias:** Presentation of predictive value of BWT/IUS parameters for future treatment response in BWT, ΔBWT, PPV/NPV, AUC
- **Moderate risk of bias:** Insufficient presentation of data.
- **High risk of bias:** Inadequate or no statistical analysis.
