# Supplementary material for: The predictive value of intestinal ultrasound for treatment response in inflammatory bowel disease: a systematic review and pooled data analysis
Source: J Crohns Colitis. 2026 Apr 15;20(4):jjag017. doi: 10.1093/ecco-jcc/jjag017 (PMC13080699; doi:10.1093/ecco-jcc/jjag017)
Supplement: jjag017_Supplementary_Data [file jjag017_supplementary_data.zip › Supplementary Table 1. Definitions of response.docx]

**Supplementary Table 1. Definitions of response**

**Crohn’s Disease**

| Author, Year | Outcome measurement | | Definition of response per included study |
| --- | --- | --- | --- |
|  | Timepoint | Type |  |
| Ainora, 2024^15^ | Week 48 | Endoscopic | Endoscopic response was defined as Simple Endoscopic Score for Crohn’s disease (SES-CD) reduction of at least 50% from baseline, whereas |
|  |  |  | Mucosal healing: was defined as SES-CD < 3. |
| Allocca, 2024^16^ | Week 52 | Endoscopic | Endoscopic remission: SES-CD ≤2 |
| Cerna, 2023^17^ | Week 30 | Therapeutic | Responder: treatment’s persistence at week 30 |
| Chen, 2022^18^ | Week 14 | Composite of clinical and endoscopic | Responder: both clinical response (ΔCDAI ≥-100 points or CDAI <150) and endoscopic response (disappearance of ulcers or a decrease of at least 50% from baseline SES-CD) |
| Dolinger, 2023^20^ | Week 52 | Endoscopic | Endoscopic remission: in the TI [SES-CD ≤ 2] |
| Dolinger, 2025^37^ | Week 26-52 | Endoscopic | Improvement: Mayo endoscopic subscore (MES) ≤1 |
|  |  |  | Response: Decrease in MES ≥1 |
|  |  |  | Remission: MES 0 |
| El-Nady, 2024^38^ | Week 12 | According to ECCO guidelines |  |
| Huang, 2024^21^ | Week 44-56 | Endoscopic | Overall Mucosal Healing a total SES-CD ≤2, while  Segmental Mucosal Healing: SES-CD of 0 and was only analyzed in segments  with SES-CD ≥3 at baseline |
|  |  | Sonographic | Transmural healing BWT ≤3.0 mm, preserved BWS, CDS 0-1, and the absence of i-fat in the most affected segment identified by IUS at baseline |
| Kucharzik, 2023^11^ | Week 48 | Endoscopic | Endoscopic response: reduction from baseline in SES-CD of ≥50%.  Endoscopic remission: was defined as SES-CD ≤2. |
| Laterza, 2021^22^ | Week 12 | Both clinical and endoscopic | Responder: a combined endoscopic and clinical outcome, defined as a decrease in SES-CD of the terminal ileum of at least 1 unit compared to baseline or SES-CD = 0 and a decrease in CDAI score ≥70 compared to baseline. |
| Lovett, 2025^23^ | Week 52 | Clinical | Responder: ≥1 point reduction in the Crohn’s disease Obstructive Symptom Score at twelve months compared to baseline. |
| Ma, 2021^32^ | >Week 52 | Steroid-free clinical remission  Drug escalation  Hospitalization (CD related) | Responder: Corticosteroid free clinical remission  Clinical remission: CDAI <150 without the use of systemic steroids or budesonide at any evaluation during follow-up.  Non-responder: need for drug escalation, need for hospitalization and need for surgery |
| Orlando, 2018^24^ | Week 52 | Therapeutic | Responder: no need for surgery |
| Paredes, 2019^29^ | Week 52 | Sonographic | Remission (Transmural healing): BWT ≤3mm and CDS grade 0 or 1  Response (Sonographic improvement): decrease in BWT of >2mm and one CDS grade |
|  | Week 210 | Therapeutic | Responder: no need for corticosteroids, no need for treatment intensification (addition of an immunomodulator, no dose increase or decreased time interval between anti-TNF doses), and no need for required surgical treatment. |
| Quaia, 2019^25^ | Week 10-18 | Endoscopic | Responder: Crohn Disease Endoscopic Index of Severity decreased from 25 to 44 before treatment to 10 to 15 after treatment based on ileum-colonoscopy combined with a histologic acute inflammatory score of less than 7 or when the CDAI score showed a  decrease of 70 or greater compared to baseline |
| Ripollés, 2008^28^ | Week 143 | Therapeutic | Responder:  Clinical remission: CDAI <150.  Therapeutic response: No need for steroids, no need for maintaining immunosuppressive therapy, no need for surgery during follow-up period  Non-responder: In the long-term analysis, an unfavorable clinical course was considered when steroids or immunosuppressive therapy had to be maintained and also when complications required surgery. |
| Ripollés, 2016^30^ | Week 12 and Week 52 | Clinical | Clinical response: HBI decrease > 3 points and CRP levels decrease, but did not restore to normal without corticosteroid use  Clinical remission: HBI < 5 and normal CRP levels without corticosteroid use  Lack of response: HBI and/or CRP increased or did not change or when other treatments were started due to disease activity |
|  |  | Sonographic | Remission: BWT ≤3mm, CDS grade 0 and absence of complications  Improvement: BWT decrease of ≥2 mm, a decrease of one CDS grade, a decrease of ≥20% of the mural enhancement, and/or disappearance of transmural complications or stenosis |
|  | Week 70 | Therapeutic | Responder: no need for corticosteroids or immunomodulators, no need for intensification of biological therapy, no complications, no need for bowel resecting surgery |
| Saevik, 2014^26^ | Week 52 | Clinical remission and therapeutic | Responder:  Clinical remission: CDAI <150 after 12 months of treatment start.  Effective treatment: was defined as continued use of same anti-TNF or discontinuation  of systemic steroids during the study period. If a patient had to change treatment due to drug’s side effects, this was not considered a treatment failure.  Non responder:  Treatment failure: a change in the drug regime >1 month after treatment start from systemic steroids to anti-TNF or from one anti-TNF to another during the follow-up period. |
| Smith, 2022^27^ | Week 46 | Therapeutic | Responder: patients who continued on prescribed therapy without an escalation or change in medical therapy, had no increase or new corticosteroid requirements from baseline, and did not require surgery, endoscopic dilatation or hospitalisation by the end of the follow-up period. |
| De Voogd, 2022^19^ | Week 12-34 | Endoscopic | Segmental endoscopic remission: SES-CD = 0  Segmental endoscopic treatment response a decrease of SES-CD ≥ 50%  Complete endoscopic remission: SES-CD = 0 in all segments. |

**Ulcerative Colitis**

| Author, Year | Treatment | Outcome measure | | Definition of response |
| --- | --- | --- | --- | --- |
|  |  | Timepoint | Type |  |
| Allocca, 2023^39^ | IFX (n=29)  ADL (n=4)  VEDO (n=14)  UST (n=2) | Average 41 weeks | Endoscopic | Endoscopic improvement: MES ≤ 1  Endoscopic remission: MES = 0 |
| Dolinger, 2025^37^ | IFX (n=12)  ADL (n=1)  USTE (n=11)  VEDO (n=8)  TOFA (n=5)  UPA (n=3)  Ozanimod (n=2 | Week 26 - 52 | Endoscopic | Endoscopic remission: EMS = 0  Endoscopic response: decrease of EMS ≥1 point  Endoscopic improvement: EMS ≤1 |
| Ollech, 2024^34^ | TOFA (n=27) | Week 8 | Clinical response | Clinical response: decrease in the fMS ≥ 3  Clinical remission: fMS of ≤2 points. |
| Parente, 2010^35^ | CS (n=74) | Week 65 | Endoscopic | Endoscopic remission: Baron score ≤1 |
| Sagami, 2022^36^ | CS n=36   Anti-TNF= 23   VEDO n=20   TOFA n=9  UST n=6   Tacrolimus n=6 | Week 8 | Clinical | Clinical Remission: PRO2 ≤1 with no rectal bleeding subscore (PRO2 remission) |
| De Voogd, 2022^6^ | TOFA (n=27) | Week 8 | Endoscopic | Endoscopic remission: EMS = 0, UCEIS = 0  Endoscopic response: decrease of EMS ≥1 point, decrease of UCEIS ≥2 points  Endoscopic improvement: EMS ≤1 |
| De Voogd, 2023^33^ | IFX n=18  ADL n=1  VEDO n=14  UST n=2  TOFA n=14  Ciclosporin n=1 | Week 8-26 | Endoscopic | Endoscopic remission: EMS = 0,  Endoscopic improvement: EMS ≤1,  Endoscopic response: a decrease of EMS ≥1.  Complete endoscopic remission and improvement: EMS = 0 or ≤1 for all segments |
| Yoshida, 2011^40^ | Cytapheresis n=26 in addition, corticosteroids,  5-asa and immunomodulator | 1 year | Clinical | Clinical remission (sustained): CDAI score of 2 or less  Clinical relapse: Score of 6 or more |

**Acute Severe Ulcerative Colitis**

| Author, Year | Treatment | Outcome measure | | Definition of response |
| --- | --- | --- | --- | --- |
|  |  | Timepoint | Type |  |
| Ilvemark, 2022^41^ | Corticosteroids iv. (n=56) | Day 7 | Clinical | Clinical responder: pMayo ≥30% and ≥3 points reduction, including a rectal bleeding subscore of 1 or 0 or a decrease in rectal bleeding subscore ≥ 1 point compared with baseline. |
|  |  |  | Therapeutic | Responder: all patients avoiding rescue therapy or surgery within the first 7 days of treatment |
| Ilvemark, 2024^43^ | Corticosteroids iv. (n=56) | 13 and 52 weeks | Therapeutic | Responder: No need for colectomy |
|  |  |  | Clinical | Clinical responder: pMayo ≥30% and ≥3 points reduction, including a rectal bleeding subscore of 1 or 0 or a decrease in rectal bleeding subscore ≥ 1 point compared with baseline. |
| Smith, 2021^31^ | Corticosteroids iv. (n=10) | Within 7 days | Therapeutic | Responder: No need for IFX salvage therapy |
| Yamagucy, 2009^42^ | Cytapheresis (n=26), concomitant:  sulfasalazine/mesalamine (n=21)  Azathioprine (n=6)  CS (n=16) | Day 7 | Clinical | Clinical Remission: CAI≤4 after treatment  Clinical response: a decrease in CAI ≥3, but CAI>4;  No response: a decrease of CAI <3 or CAI was increased or not  reduced by the treatment |
