# Supplementary material for: The predictive value of intestinal ultrasound for treatment response in inflammatory bowel disease: a systematic review and pooled data analysis
Source: J Crohns Colitis. 2026 Apr 15;20(4):jjag017. doi: 10.1093/ecco-jcc/jjag017 (PMC13080699; doi:10.1093/ecco-jcc/jjag017)
Supplement: jjag017_Supplementary_Data [file jjag017_supplementary_data.zip › Supplementary Table 2. Ultrasound scores.docx]

**Supplementary Table 2. Ultrasound scores**

| **Author, year** | **Ultrasound score** |
| --- | --- |
| Allocca, 2023^39^  Dolinger, 2025^37^  Ollech, 2024^34^ | Milan Ultrasound Criteria (MUC)  MUC = 1.4 × BWT [mm] + 2 × CDS; (where CDS = 1 if present, or CDS = 0 if absent) |
| Allocca, 2024^16^ | Bowel ultrasound Score (BUSS)  BUSS = 0.75 x BWT + 1.65 x CDS; (where CDS = 1 if present, or CDS = 0 if absent). |
| Cerna, 2023^17^ | IUS score:  Definition unknown: Intestinal ultrasound score was determined with the expertise developed in the STARDUST clinical study according to Kucharzik |
| Dolinger, 2023^20^  Dolinger, 2025^37^  Huang, 2024^21^ | The International Bowel Ultrasound Segmental Activity Score (IBUS-SAS)[0-100]:  IBUS-SAS = 4*BWT + 15*inflammatory fat presence + 7*CDS+ 4*bowel wall stratification) |
| Dolinger, 2025^37^ | Civitelli index [0-4]: BWT >3mm (yes/no) + CDS present (yes/no) + BWS absent (yes/no) + haustrae absent (yes/no) |
| Parente, 2010^35^ | Ultrasound score:  US severity was graded 0 – 3 according to the maximum BWT and the degree of CDS  Grade 0: BWT < 4 mm and no or scarce CDS;  Grade 1: BWT 4 – 6 mm and CDS  Grade 2: BWT 6 – 8 mm and CDS  Grade 3: BWT > 8 mm and CDS |
