# Supplementary material for: The predictive value of intestinal ultrasound for treatment response in inflammatory bowel disease: a systematic review and pooled data analysis
Source: J Crohns Colitis. 2026 Apr 15;20(4):jjag017. doi: 10.1093/ecco-jcc/jjag017 (PMC13080699; doi:10.1093/ecco-jcc/jjag017)
Supplement: jjag017_Supplementary_Data [file jjag017_supplementary_data.zip › 12.docx]

**Supplementary Figure 1. ROC curves of different outcomes at week 12 - 16 for anti-TNF treated CD patients**

Clinical (n=51): AUC 0.628 (p=0.160) Δ-30% BWT (sens 38%, spec 93%)

Therapeutic (n=36): AUC 0.761 (p=0.026) Δ-11% BWT (sens 64%, spec 88%)

Endoscopic (n=84): AUC 0.919 (p<0.001) Δ-15% BWT (sens 90%, spec 85%)
