# Supplementary material for: The predictive value of intestinal ultrasound for treatment response in inflammatory bowel disease: a systematic review and pooled data analysis
Source: J Crohns Colitis. 2026 Apr 15;20(4):jjag017. doi: 10.1093/ecco-jcc/jjag017 (PMC13080699; doi:10.1093/ecco-jcc/jjag017)
Supplement: jjag017_Supplementary_Data [file jjag017_supplementary_data.zip › Supplementary Material 1. Search terms in PubMed.docx]

**Supplementary Material 1. Search terms in PubMed**

The following search terms were included: ("Inflammatory Bowel Diseases"[Mesh] OR inflammatory bowel disease*[tiab] OR IBD[tiab] OR crohn*[tiab] OR colitis ulcer*[tiab] OR ulcerative colit*[tiab] OR ileitis[tiab] OR colitis[tiab] OR proctocolitis[tiab]) AND ("Ultrasonography"[Mesh] OR ultraso*[tiab] OR sonograph*[tiab] OR bowel ultraso*[tiab] OR intestinal ultraso*[tiab] OR transabdominal ultraso*[tiab] OR bowel sono*[tiab] OR intestinal sono*[tiab] OR IUS[tiab] OR SBUS[tiab] OR echograph*[tiab] OR pocus[tiab] OR point-of-care ultrasound*[tiab]) AND ("Predictive Value of Tests"[Mesh] OR "Disease-Free Survival"[Mesh] OR "Treatment outcome"[Mesh] OR predict*[tiab] OR respons* [tiab] OR probabilit*[tiab] OR long term outcome*[tiab] OR clinical outcome*[tiab]) NOT ("Congress" [Publication Type] OR "Clinical Conference" [Publication Type] OR "Letter" [Publication Type] OR "Editorial" [Publication Type] OR letter[ti] OR editorial[ti]).
